# Nanocarriers Derived from *Annona squamosa* Seed Oil Amplify the Anti-inflammatory Effect of Carvacrol in Human Neutrophils

**DOI:** 10.1021/acsomega.5c12743

**Published:** 2026-02-17

**Authors:** Sarah Brenda Ferreira dos Santos, Stéfano Arrais Pereira, Maria Júlia Pereira dos Santos, Louhana Moreira Rebouças, Francisco Alessandro Marinho Rodrigues, João Francisco Câmara Neto, Matheus da Silva Campelo, Luzia Kalyne Almeida Moreira Leal, Francisca Rayssa Mesquita, Denise Ramos Moreira, Victor Borges Fernandes, Ícaro Gusmão Pinto Vieira, Nágila Maria Pontes Silva Ricardo

**Affiliations:** † Laboratory of Polymers and Materials Innovation, Department of Organic and Inorganic Chemistry, Sciences Center, 28121 Federal University of Ceara, Campus of Pici, 60440-900 Fortaleza, Ceará, Brazil; ‡ Department of Pharmacy, Universidade Federal do Ceará, Rua Capitão Francisco Pedro, 1210, 60430-170 Fortaleza, Ceará, Brazil; § Technological Development Park, Universidade Federal do Ceará, Campus of Pici, 60440-900 Fortaleza, Ceará, Brazil

## Abstract

This research aimed to prepare nanoemulsions based on *Annona squamosa* seed oil (ASSO) for encapsulation of carvacrol (CARV) and subsequent evaluation of its cytotoxicity and anti-inflammatory potential in human neutrophils. The chemical composition of ASSO is rich in long-chain fatty acids, especially oleic and linoleic acids. The treatment of human neutrophils with ASSO demonstrated its low cytotoxicity (1–50 μg mL^–1^), as well as anti-inflammatory effect at 25 and 50 μg mL^–1^, being able to attenuate the release of myeloperoxidase (MPO). The nanocarriers presented colloidal stability with particle sizes around 170 nm, ζ-potential greater than |30 mV| and moderate polydispersity. The encapsulation efficiency of the nanosystems was greater than 99%, evidencing the effectiveness of the applied methodology for CARV entrapment. The drug release tests demonstrated that the nanoemulsions were able to prolong the carvacrol release process, with an accumulated release of 26% (745 μg) in 72 h, with the Korsmeyer–Peppas model being the one that best adjusted to the observed kinetics. CARV, carvacrol-loaded nanoemulsion (CNE), and blank nanoemulsion (BNE) showed low cytotoxicity (5–100 μg mL^–1^) against human neutrophils and were able to reduce neutrophil degranulation. Notably, the CNE potentiated the anti-inflammatory effect of the CARV, demonstrating biological efficacy at lower concentrations (5 μg mL^–1^) compared to the free drug (50 μg mL^–1^). Thus, nanoemulsions based on ASSO were effectively enhanced the biological effects of CARV, positioning themselves as promising nanosystems for the encapsulation and delivery of lipophilic compounds.

## Graphical abstract

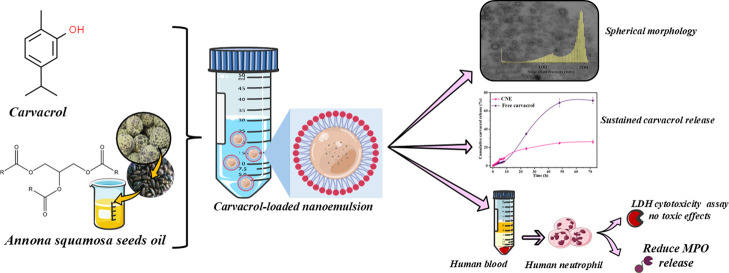

## 1 Introduction

Inflammatory responses constitute essential components of innate immunity, playing a central role in pathogen elimination and the restoration of tissue homeostasis.^1^ This process involves the recruitment of neutrophils to the site of injury, where these cells contribute to the initiation of tissue repair through phagocytosis and the release of inflammatory mediators and proteolytic enzymes.^2^ However, dysregulated or persistent inflammatory activation may progress to chronic inflammation, thereby contributing to the development of several inflammatory and immune-mediated diseases, including rheumatoid arthritis, systemic lupus erythematosus, psoriasis, inflammatory bowel disease, and cardiovascular disorders.^3,4^

The clinical management of these conditions relies primarily on the use of glucocorticoids, steroids, and nonsteroidal anti-inflammatory drugs (NSAIDs).^5^ Although widely prescribed, these medications present significant limitations, such as nonspecific biodistribution, reduced bioavailability and the recurrence of side effects, including renal, gastrointestinal and cardiovascular toxicity.^6,7^ Given these limitations, the search for safer and more effective therapeutic alternatives becomes essential.

Natural compounds have emerged as promising candidates for the treatment of inflammatory diseases due to their broad pharmacological spectrum and lower incidence of side effects.^8^ One of the compounds that has aroused great interest is carvacrol (5-isopropyl-2-methylphenol), a phenolic monoterpene presents in plants of the Lamiaceae family.^9^ Studies have demonstrated that this bioactive compound exhibits antioxidant, antimicrobial, antiviral, and hepatoprotective properties, in addition to exerting a modulatory effect on pro-inflammatory cytokines.^10,11^ Furthermore, carvacrol is classified as Generally Recognized as Safe (GRAS) and is approved by the Food and Drug Administration (FDA) for food use.^12^ However, its therapeutic use is limited by unfavorable physical and chemical characteristics, such as low solubility in water and high volatility.^13^

Nanostructured drug delivery systems have been extensively investigated as a strategy to overcome these limitations.^14^ Among them, nanoemulsions (NEs) stand out due to their high encapsulation efficiency, controlled release profile, and ability to protect bioactive compounds against degradation and volatilization.^15^ NEs are colloidal systems stabilized by surfactants, capable of forming droplets at the nanometric scale, thereby conferring kinetic stability to the formulations.^16^ In the present study, Pluronic F127, a biocompatible amphiphilic triblock copolymer, was employed as a stabilizing agent owing to its ability to prolong the systemic circulation of nanoparticles by reducing their rapid uptake by macrophages.^17^

Despite their wide applicability, essential oil-based nanoemulsions are particularly susceptible to Ostwald ripening, one of the main destabilization mechanisms affecting these systems.^18^ The incorporation of medium- or long-chain triglycerides into the oil phase has proven to be an effective strategy to mitigate this phenomenon.^19^ In this context, oil extracted from *Annona squamosa* Linn. seeds emerges as a promising alternative. This species, belonging to the Annonaceae family, is widely distributed in tropical and subtropical regions and holds considerable economic and nutritional relevance.^20^

The seeds, which account for approximately 30% of the fruit mass, are frequently discarded as agro-industrial waste.^21^ However, they represent a rich source of long-chain fatty acids, particularly oleic and linoleic acids, which contribute to the colloidal stability of nanoemulsions containing essential oils.^22^ In addition, several studies recognize the therapeutic potential of this vegetable oil, which includes antioxidant activity,^20^ antitumor^23^ and anti-inflammatory^24^ properties. However, research focused on its application in nanostructured formulations is still scarce.

Considering the above, the present study aimed to develop and characterize oil-in-water nanoemulsions based on *A. squamosa* seed oil for the encapsulation of carvacrol and to evaluate the influence of this oil on the anti-inflammatory properties of the encapsulated carvacrol. The formulations were evaluated in terms of physicochemical stability, encapsulation efficiency, and release profile. Furthermore, clinical safety assays and anti-inflammatory activity evaluations were conducted using human neutrophils as a biological model. The proposed system seeks to provide a biocompatible nanoplatform capable of enhancing the therapeutic performance of carvacrol by mitigating the limitations associated with its physicochemical instability.

## 2 Experimental Section

### 2.1 Materials

The seeds of *A. squamosa* L. used as raw material in the present study were collected in the municipality of Pacoti—Ceará. The use of this species was registered in the National System for Management of Genetic Heritage and Associated Traditional Knowledge (SisGen) under registration number AF8D0A2. All reagents used in this study were provided by Sigma-Aldrich. Carvacrol (5-isopropyl-2-methylphenol) CAS number 499-75-2. Pluronic F127 CAS number 9003-11-6. Acetonitrile CAS number 75-05-8. PMA (phorbol-12-myristate-13-acetate) CAS NUMER 16561-29-8, Triton X-100, CAS number 9036-19-5. Deionized water was obtained from a Milli-Q Millipore Corporation purification system (Watford, United Kingdom). All reagents used in the research were of analytical grade.

### 2.2 Extraction of *A. squamosa* Seed Oil (ASSO)

The vegetable oil in the preparation of nanoemulsions was obtained following a protocol proposed by dos Santos et al. (2025).^21^ Seeds from *A. squamosa* fruits were first washed, dried, and mechanically ground. Approximately 100 g of the processed material was placed into a filter paper envelope and inserted into a Soxhlet apparatus, to which 500 mL of hexane was added. The extraction was carried out under reflux at 60 °C for 6 h. After completion, the organic phase was dried over anhydrous sodium sulfate (Na_2_SO_4_) to remove residual moisture and then concentrated using a rotary evaporator under vacuum. The recovered oil was then subjected to oven at 105 °C for 1 h to eliminate any remaining traces of water. The entire extraction procedure was performed in triplicate.

### 2.3 Gas Chromatography–Mass Spectrometry

The fatty acid profile of *A. squamosa* seed oil (ASSO) was determined by gas chromatography–mass spectrometry (GC–MS) analysis after hydrolysis and conversion to methyl esters. Analyses were carried out on an Agilent 8890 GC system coupled to a gas chromatograph/mass spectrometry detector (GC/MSD, model 5977 B, Agilent) using the DB-5 column (95% dimethylpolysiloxane, 5% phenylpolysiloxane), J&W Scientific (now Agilent), 18 m × 0.25 mm, film thickness d*F* = 0.1 μm, temperature gradient: 80 °C (3 min isotherm), 10 °C min^–1^ to 320 °C (10 min isotherm); carrier gas: He (1.1 mL min^–1^), split injection at 280 °C.

### 2.4 Preparation of Nanoemulsions

The nanoemulsions referred to as BNE (control nanoemulsion) and CNE (carvacrol-loaded nanoemulsion) were synthesized according to the emulsification method proposed by.^25^ For the preparation of the formulations, the immiscible phases were first prepared and labeled as aqueous phase and organic phase. The aqueous phase was produced by dissolving 300 mg of Pluronic F127 in 9.25 g of Milli-Q water. The organic phase was prepared by dissolving 30 mg of carvacrol in 750 mg of fixed oil extracted from *A. squamosa* L. seeds. After preparing the immiscible phases, the aqueous phase was added to the organic phase, and the mixture was subjected to a homogenization process at 500 rpm for 1 h. After the established time, the formulation obtained was subjected to ultrasonic irradiation on a Branson Sonifier W-450D (Hielsher, Teltow, Germany) with probe, at amplitude of 70% and 100–105 W power, for 3 min in 18 cycles of 10 s on and 10 s off, into an ice bath.

### 2.5 Characterization of Nanoemulsions

#### 2.5.1 Dynamic Light Scattering (DLS)

The stability parameters of the prepared nanoemulsions were evaluated in terms of particle size, polydispersity index (PDI), and zeta potential. The analysis was conducted using a Zetasizer Nano ZS model ZEN 3600 (Malvern Instruments, UK). For the experiment, the samples were diluted in deionized water at a ratio of (1:1000) (v v^–1^) and analyzed at a fixed angle of 90° and at 25 °C.^26^ The results of hydrodynamic diameter and polydispersity index, as well as the surface charge of the samples were expressed as an average of five measurements.

#### 2.5.2 Thermodynamic and Storage Stability Studies

Thermodynamic stability tests were performed to identify the presence of metastable formulations. The carvacrol-loaded formulation (CNE) was subjected to thermal and mechanical stress conditions to evaluate their effects on the physicochemical properties of the system, in accordance with the methodology described by Md et al., (2020).^27^ Mechanical stress was assessed by centrifugation at 10,017*g* for 30 min. Thermal stress was evaluated using two temperature variation protocols. In the first test, samples were subjected to six consecutive heating–cooling cycles between 45 and 4 °C, with a storage period of 48 h at each temperature. In the second protocol, samples were exposed to three freeze–thaw cycles, with temperatures ranging from −21 to 25 °C and a holding time of 48 h under each condition. After each test, the formulations were visually inspected for evidence of phase separation or precipitation.

The long-term storage stability tests were conducted over a period of 90 days to assess the macroscopic aspects and physicochemical parameters of the CNE sample. For this purpose, the freshly prepared formulations were placed in hermetically sealed glass tubes with a capacity of 10 mL. The samples were stored in an incubator at 25 ± 2 °C and analyzed at intervals of 1, 15, 30, 60, and 90 days. Colloidal stability parameters, including particle size, polydispersity index, and ζ-potential, were investigated, with analyses performed in quintuplicate.^17,18^

#### 2.5.3 Morphology Analyses

The morphology of the nanodroplets (CNE and BNE) was evaluated using scanning transmission electron microscopy (STEM) and confocal laser scanning microscopy (CLSM). STEM analyses were performed using a Tescan Vega 3 electron microscope (Tescan, Brno, Czech Republic) operating at an acceleration voltage of 30 kV. The size histogram was assessed by measuring 100 randomly selected particles with the aid of the ImageJ software. STEM was carried out as previously described by (Liew et al., 2020)^28^, where initially, 50 μL of the formulations were diluted in deionized water at a ratio of 1/150 (v v^–1^) and deposited on a carbon-coated copper grid (Formvar mesh over 200 copper grids). The samples were then fully dried overnight at room temperature.

The CLSM analyses were conducted following the methodology described by,^29^ with adaptations. For staining the CNE formulation, 20 μL of Nile red solution in chloroform (1 mg mL^–1^) were added to 1 mL of the sample. The mixture was homogenized in the absence of light to ensure complete staining of the oil droplets in the formulation.

After the staining procedure, a 20 μL aliquot of the stained sample was placed on a glass slide and covered with a coverslip. The analysis was performed using a Zeiss LSM-710 confocal microscope (Leica, Heidelberg, Germany), with excitation at 543 nm, a wavelength specific to the dye, using a continuous-wave argon ion laser. Images were captured using a 40× oil immersion objective lens and processed with the LSM 710 ZEN software associated with the analytical instrument.

#### 2.5.4 Encapsulation Efficiency (% EE) and Drug Loading Capacity (% DLC)

The quantification of carvacrol in the CNE formulation was performed using high performance liquid chromatography (HPLC) to determine the encapsulation efficiency (% EE) and drug loading capacity (% DLC). The analyses were conducted under isocratic elution mode on a Shimadzu LC-10AD chromatograph equipped with a UV–vis diode array detector SPD-M10AVP, utilizing a C18(2) column (5 μm, 4.6 × 150 mm) with detection at 274 nm. The mobile phase used consisted of acetonitrile/water (50:50, v v^–1^) at a flow rate of 1.0 mL min^–1^, with a run time of 15 min and an injection volume of 20 μL. The standard curve was prepared by dissolving 10 mg of carvacrol in 10 mL of an acetonitrile and deionized water mixture in an 80:20 (v v^–1^) ratio. Six standards were prepared, and the concentration range evaluated was from 10 μg mL^–1^ to 1000 μg mL^–1^. All samples were filtered prior to injection (0.45 μm filters, Merck, Darmstadt, DE).^30^

For the determination of EE %, 1 mL aliquots of CNE were centrifuged in Turbo 15 Vivaspin ultrafiltration tubes with a 3000 Da cutoff (Sartorius, Göttingen, Germany) at 4000*g* for 30 min. The encapsulation efficiency and drug loading capacity of carvacrol were determined by the difference between the initial concentration of carvacrol added to the formulation and the amount of free carvacrol analyzed after the filtration process. EE % and DLC % were expressed as percentages and deduced according to the eqs 1 and 2, respectively

1 $$\%\;EE=\frac{CNE-CF}{CNE}\times 100$$

2 $$\%\;DLC=\frac{W_{totalcarv}-W_{freecarv}}{W_{NE}}\times 100$$

where CTNE corresponds to the initial concentration of carvacrol present in CNE (μg mL^–1^), CF to the concentration of the filtered material (μg mL^–1^), *W*_totalcarv_ corresponds to the weight of the drug used in the preparation of the nanoemulsion, *W*_freecarv_ represents the mass of free drug detected in the supernatant after the ultrafiltration process and *W*_NE_ represents the total mass of the formulation.

### 2.6 In Vitro Drug Release and Kinetic Studies

#### 2.6.1 Determination of Sink Conditions

To investigate the release profile of carvacrol in its free and encapsulated forms within the CNE formulation, solubility assays of the active compound were initially conducted using different proportions of a receptor medium composed of a mixture of ethanol and phosphate buffer (PBS, pH 7.4), aiming to establish sink conditions. The tested proportions in this study were 10/90, 20/80, 25/75, and 30/70 (ethanol/PBS, pH 7.4). In this procedure, an excess amount of carvacrol was added to flasks containing 10 mL of the receptor medium. The suspensions were subjected to magnetic stirring at 500 rpm at room temperature for 2 h, followed by absorbance measurements of the solutions. The sink condition was defined based on the proportion in which the dissolution volume exhibited a carvacrol solubility at least three times greater than the corresponding saturation volume.^31,32^ The experimental data obtained are presented in Table 3.

#### 2.6.2 In Vitro Drug Release Assays

The drug release assay was performed to evaluate the release profile of carvacrol-loaded nanoemulsions.^33,34^ For this purpose, samples of unencapsulated carvacrol (CARV) and carvacrol-loaded nanoemulsions (CNE) were analyzed. The experiments were carried out at 37.0 ± 0.5 °C for 72 h in a double-compartment system separated by a dialysis membrane (MWCO 12–14 kDa, Sigma-Aldrich, St. Louis, USA) with a permeation area of 1.09 ± 0.03 cm^2^. The donor compartment was filled with 1 mL of sample (CNE or CARV), while the receptor compartment was filled with 12 mL of a phosphate buffer mixture (pH 7.4) and ethanol, under constant magnetic stirring at 500 rpm. The assays were performed in triplicate (for each sample) and conducted under *sink* conditions. For carvacrol quantification, 1 mL aliquots were collected from the receptor compartment at predetermined time intervals and analyzed using a UV–vis spectrophotometer (2600 Shimadzu) at 274 nm. The carvacrol concentration was determined using a calibration curve (*A*_274_ = 0.015­[CARV] + 0.0034), obtained from a fit (*R* ^2^ = 0.9998) measured for different carvacrol dilutions (from 1 to 50 μg mL^–1^).

The release kinetics were determined according to the mathematical models of zero-order,^35^ first-order,^36^ Higuchi,^37^ and Korsmeyer–Peppas,^38^ following eqs 3–6, respectively

3 $$zero‐order:Q=k_{0}t+Q_{0}$$

4 $$first‐order:\ln Q={\ln Q}_{0t}+k_{1}t$$

5 $$Higuchi:Q=k_{H}t^{1/2}$$

6 $$\mathrm{Korsmeyer-Peppas}:\frac{M^{t}}{M^{\infty }}=k_{\mathrm{KP}}t^{n}$$

where, *Q* represent the cumulative amount of drug released at time *t*, *Q*_0_ corresponds the initial amount of drug in solution, *M* ^*t*^/*M* ^∞^ is the fraction of the drug released at time *t*, *n* is the diffusion exponent, *k*_0_, *k*_1_, *k*_H_ and *k*_KP_ are the release constants for the zero-order, first-order, Higuchi model and Korsmeyer–Peppas, respectively.

### 2.7 Preclinical Assessment of Safety and Efficacy Using Human Neutrophils

#### 2.7.1 Human Blood Samples

The experiments were conducted in accordance with the guidelines of Resolution 466/2012 of the National Health Council. Accordingly, this study was submitted to and approved by the Research Ethics Committee of the Federal University of Ceará, under protocol CAAE: 86014724.0.0000.5054. For the experimental protocol, the samples evaluated were: CARV (unencapsulated carvacrol), CNE (carvacrol-loaded nanoemulsion), and BNE (nanoemulsion without the encapsulated drug, produced to be used as a control). For the preclinical assay, peripheral blood from healthy donors was collected on the day of the experiment by the team from the Center for Pharmaceutical and Cosmetic Studies at the Federal University of Ceará, Fortaleza, Brazil. The material was placed in tubes containing 0.8% sodium citrate (w v^–1^) and centrifuged at 1250*g* for 10 min to separate the blood components. Neutrophils were isolated according to the methodology proposed by,^39^ resulting in a cell suspension containing 80–90% neutrophils with 90 ± 2.0% viability, obtained through the Trypan Blue exclusion assay.

#### 2.7.2 Lactate Dehydrogenase (LDH) Assay

Human neutrophils in suspension (2.5 × 10^6^ cells mL^–1^) were incubated with the active compounds ASSO (1–50 μg mL^–1^), CARV (5–100 μg mL^–1^), CNE (5–100 μg mL^–1^), and BNE (dilution equivalent to a concentration of 100 μg mL^–1^ of CNE), vehicle (0.1% DMSO, negative control), Hanks’ balanced salt solution (HBSS, untreated cells), and Triton X-100 (0.2% v v^–1^, cytotoxic standard) at 37 °C for 15 min in a 96-well plate. After incubation, samples were centrifuged at 800*g*, 4 °C for 10 min. Supernatants were separated and transferred to a new 96-well plate. Absorbance was then measured at 340 nm and LDH enzyme activity was assessed according to the manufacturer’s instructions (LDH liquiform from Labtest Diagnóstica, Lagoa Santa, MG, Brazil). LDH activity was calculated according to eq 7

7 $$A=\left[\frac{\left(A_{1}-A_{2}\right)}{2}\right]\times 1746.03$$

where *A* corresponds the LDH enzyme activity (U L^–1^), *A*_1_ represents the absorbance at 1 min, *A*_2_ is the absorbance at 3 min and 1746.03 represents the correction factor calculated by the manufacturer (for 25 μL).^40^

#### 2.7.3 Myeloperoxidase (MPO) Release Assay

A neutrophil suspension (5.0 × 10^6^ cells mL^–1^) was incubated with the active components: ASSO (1–50 μg mL^–1^), CARV (5–100 μg mL^–1^), CNE (5–100 μg mL^–1^), and BNE (at dilutions equivalent to 5–100 μg mL^–1^ of CNE), DMSO (vehicle) and HBBS (untreated cells), indomethacin (36 μg/mL, anti-inflammatory standard) for 30 min at 37 °C. Subsequently, excluding the Hanks group, the incubated cells were stimulated by adding 0.1 μmol L^–1^ of PMA for 15 min at 37 °C. Then, the samples were centrifuged (2000*g*) at 4 °C for 15 min, and the supernatant obtained, rich in enzymes released by leukocyte degranulation, was used to determine the concentration of myeloperoxidase (MPO), according to the methodology described by Campelo et al., (2024).^41^

For the quantification of MPO concentration, phosphate buffer (pH 7.4) and hydrogen peroxide (H_2_O_2_, 0.012% v v^–1^) were added to the supernatant, and the samples were incubated at 37 °C for 5 min. After this period, 1.5 mmol L^–1^ of 3,3′,3,5′-tetramethylbenzidine (TMB) was added to the reaction mixture, and the reaction was stopped by the addition of sulfuric acid (H_2_SO_4_, 4.0 mol L^–1^). The procedure described above was performed in triplicate, and sample readings were taken at an absorbance of 595 nm.

### 2.8 Statistical Analysis

Results were expressed as the mean ± SEM of at least three independent experiments. The evaluation of data normality was performed using the Shapiro–Wilk test. The results that presented a parametric distribution were analyzed by one-way ANOVA using the Tukey test as post hoc. While the data that presented a nonparametric distribution were analyzed using the Kruskal–Wallis test followed by the Dunn’s post-test. The significance level was set at *p* < 0.05. The analyses were carried out using the statistical software GraphPadPrism 6.0 (USA).

## 3 Results and Discussion

### 3.1 Chemical Characterization of *A. squamosa* Seed Oil

The present study aimed to develop nanoemulsions using the oil extracted from the seeds of *A. squamosa* L. (ASSO) as the organic phase. This oil, obtained and characterized by its fatty acid profile as described by dos Santos et al., (2025),^21^ had an average extraction yield of 33 ± 2.3% (w/w) in relation to the mass of crushed seeds. To determine the fatty acid composition, the ASSO was subjected to a chemical modification process that initially involved a hydrolysis reaction followed by derivatization, aiming at obtaining the respective methyl esters. The analysis was performed by gas chromatography coupled with mass spectrometry (GC–MS), and the results are presented in Table 1.

**Table 1:** Chemical Composition of Fatty Acids Presents in *Annona squamosa* L. Seed Oil

Retention time (min)	Compounds	Molecular formula	Composition (%)
17.300	palmitic acid	C_16_H_32_O_2_	18.10
18.961	linoleic acid	C_18_H_32_O_2_	21.10
19.036	oleic acid	C_18_H_34_O_2_	43.60
19.246	stearic acid	C_18_H_36_O_2_	15.80
21.974	eicosanoic acid	C_20_H_40_O_2_	1.40

As shown in Table 1, ASSO is composed of five distinct fatty acids. The saturated fraction is predominantly composed of palmitic (18.10%), stearic (15.80%), and eicosanoic (1.40%) acids, while the unsaturated fraction is mainly formed by oleic (43.10%) and linoleic (21.10%) acids. The fatty acid profile identified in this study is consistent with previously reported literature data and exhibits behavior similar to that observed by Cheng et al., (2016)^23^ who employed an extraction methodology analogous to that used in this study to obtain this vegetable oil.

In the context of preparing nanoemulsions for the encapsulation of essential oils, the incorporation of vegetable oils rich in long-chain fatty acids into the dispersed phase plays a decisive role in system stability. This is because such colloidal systems are particularly susceptible to destabilization mechanisms, especially Ostwald ripening, a phenomenon characterized by the progressive increase in droplet radius accompanied by a reduction in the total number of dispersed droplets.^42^ This process is driven by differences in Laplace pressure, which is higher in smaller droplets than in larger ones, thereby favoring the diffusion of material from the dispersed phase.^43^

In this regard, triacylglycerol molecules present in the vegetable oil act as inhibitors of Ostwald ripening, contributing to the formation of kinetically more stable formulations.^19^ Furthermore, ASSO is predominantly composed of long-chain fatty acids, a feature that is particularly desirable for physiological applications, as these fatty acids exhibit lower susceptibility to metabolic degradation compared to medium-chain fatty acids. This characteristic enhances the potential of ASSO for the development of nanoengineered systems with therapeutic purposes.^44^

### 3.2 Cytocompatilibity of *A. squamosa* Seed Oil against Human Neutrophils

Lactate dehydrogenase (LDH) is an essential cytoplasmic enzyme that plays a fundamental role in the anaerobic glycolytic pathway.^45^ The release of this enzyme into the extracellular medium, detectable in the blood, serves as an indicator of cytoplasmic membrane rupture, which may be associated with several pathological conditions such as anemia, liver diseases, cardiopathies, and infectious processes. Thus, LDH is widely used as a marker of tissue damage, and its activity assay serves as a tool to investigate the potential cytotoxic effects of the compound under study.^41^

The treatment of human neutrophils with TX (cytotoxic standard) significantly increased (*p* < 0.05) LDH activity compared to the HBSS group (negative control) (Figure 1). In contrast, there was no statistically significant difference (*p* > 0.05) between the treatment of these cells with ASSO and HBSS group, indicating its low cytotoxic potential in the concentration range under study (1–50 μg mL^–1^). These findings are important because they demonstrate the safety of this fixed oil, from the seeds of *A. squamosa* L. fruits, widely used in food preparations, such as candies, ice cream and juices.^20^

**Figure 1:**
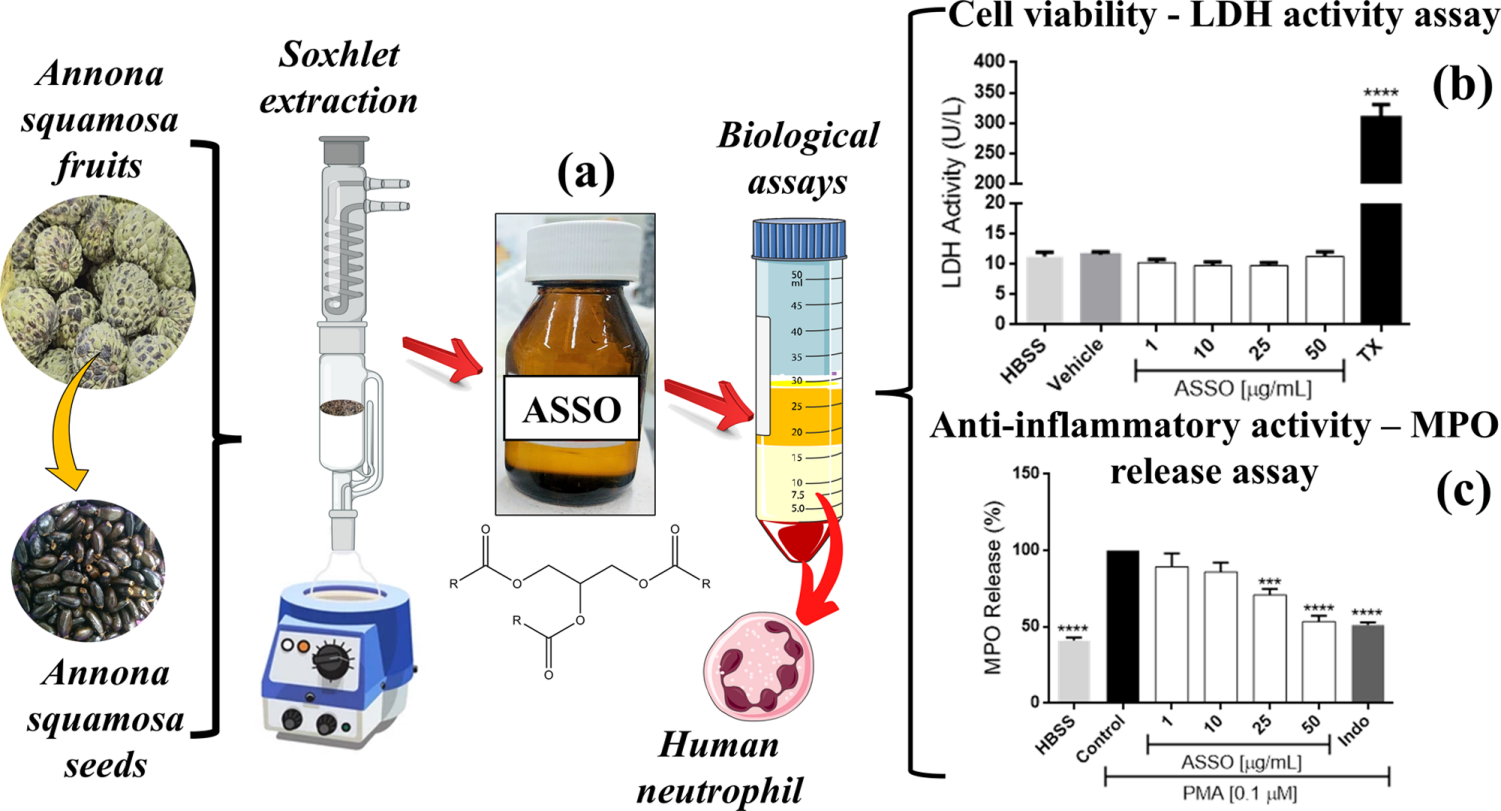
Extraction and cytocompatilibity of *Annona squamosa* seeds oil: (a) ASSO extraction; (b) ASSO LDH assay and (c) ASSO MPO release assay.

The inflammatory response in the human body is closely associated with the recruitment of defense cells during infectious processes.^46^ In this context, neutrophils are the first cells to be activated in the immune system. Among the enzymes produced by neutrophils during the immune response, myeloperoxidase (MPO) stands out for its crucial role in converting hydrogen peroxide into hypochlorous acid, a powerful endogenous oxidizing agent. Thus, MPO activity is often used as a parameter to evaluate the anti-inflammatory capacity of bioactive compounds.^47^

To perform the test in question, PMA (phorbol-myristate-acetate), an ester analogous to diacylglycerol (DAG), was used with the aim of inducing the inflammatory process. This compound can penetrate the plasma membrane and directly activate different isoforms of protein kinase C (PKC) in neutrophils. PKCs, in turn, promote the phosphorylation of cytosolic components of NADPH oxidase, facilitating the assembly of this complex in the plasma membrane and specific granules. As a result, reactive oxygen species are formed. Under PMA stimulation, neutrophils intensify the inflammatory response through the release of myeloperoxidase (MPO), a process mediated by degranulation.^41,48^

Exposure of human neutrophils to ASSO at 25 (70.9 ± 3.9%) and 50 μg mL^–1^ (53.39 ± 3.8%) reduced (*p* < 0.05) PMA-induced neutrophil degranulation, indicating that this fixed oil has anti-inflammatory activity and can attenuate MPO release. It is worth mentioning that the results obtained for ASSO at 50 μg mL^–1^ were similar to those observed for indomethacin (51.26 ± 1.7%), a nonsteroidal anti-inflammatory drug used to treat different clinical conditions in which the inflammatory component plays a key role. These findings corroborated with Bhoir et al., (2019)^24^ who reported the biological potential of ASSO in inflammatory processes, including a significant reduction in the levels of pro-inflammatory cytokines such as IL-6, IL-17, TNF-α, INF-γ, as well as a decrease in CD4^+^ T cell infiltration in psoriasis models, a chronic inflammatory condition.

Based on these results, it is estimated that ASSO is a safe and effective plant derivative for pharmaceutical applications, where it can be used as a plant-based active pharmaceutical ingredient or as emollient in the development of dispersed systems, such as emulsions. As an active ingredient, ASSO can be used in the formulation of functional foods or in the preparation of herbal medicines for the treatment of inflammatory conditions. Furthermore, due to its composition rich in unsaturated fatty acids, oral administration can be advantageous. In this study, ASSO was used in the development of nanoemulsions with anti-inflammatory potential, due to its low cytotoxicity against human neutrophils and its ability to attenuate neutrophil degranulation.

### 3.3 Physicochemical Characterization of Nanoemulsions

The colloidal stability of the prepared formulations was evaluated based on particle size, polydispersity index (PDI), and ζ-potential parameters, using the dynamic light scattering (DLS) and electrophoretic mobility techniques. The obtained results are summarized in Table 2.

**Table 2:** Characterization of Synthesized Nanoemulsions (Mean ± SD, *n* = 3)

	Analysis					
	DLS					
Sample	*D*_h_ (nm)	PDI	ζ potential (mV)	EE %	DLC %	Drug release (%)
CNE^a^	172 ± 1.7	0.1 ± 0.03	–32 ± 2.6	99.3 ± 0.06	9.9 ± 0.0006	26.2 ± 2.2
BNE^b^	169 ± 0.8	0.1 ± 0.01	–34 ± 0.9	–	–	–

a Carvacrol-loaded nanoemulsion.

b Unloaded nanoemulsion (blank control).

As shown in Table 2, the CNE and BNE formulations exhibited hydrodynamic diameters ranging from 169 to 172 nm. In colloidal systems, the formation of nanometric droplets enhances kinetic stability, as Brownian motion surpasses gravitational forces, providing greater resistance to sedimentation.^49^ Furthermore, smaller particles have a higher surface area, which may improve interactions within the physiological environment.^50^

The particle size distribution profile (Figure 2) was assessed through PDI values, a parameter that reflects the system’s heterogeneity. Colloids can be classified as monodisperse (PDI < 0.1), moderately polydisperse (0.1 < PDI < 0.3), or polydisperse (PDI > 0.4).^51^ PDI values close to 0.1 indicate a uniform droplet distribution, resulting in greater stability against destabilization phenomena. In this study, the PDI values for the BNE and CNE samples were 0.10 and 0.13, respectively. Although these indicate moderate polydispersity, both values are close to 0.1, suggesting satisfactory stability of the tested formulations.^52^

**Figure 2:**
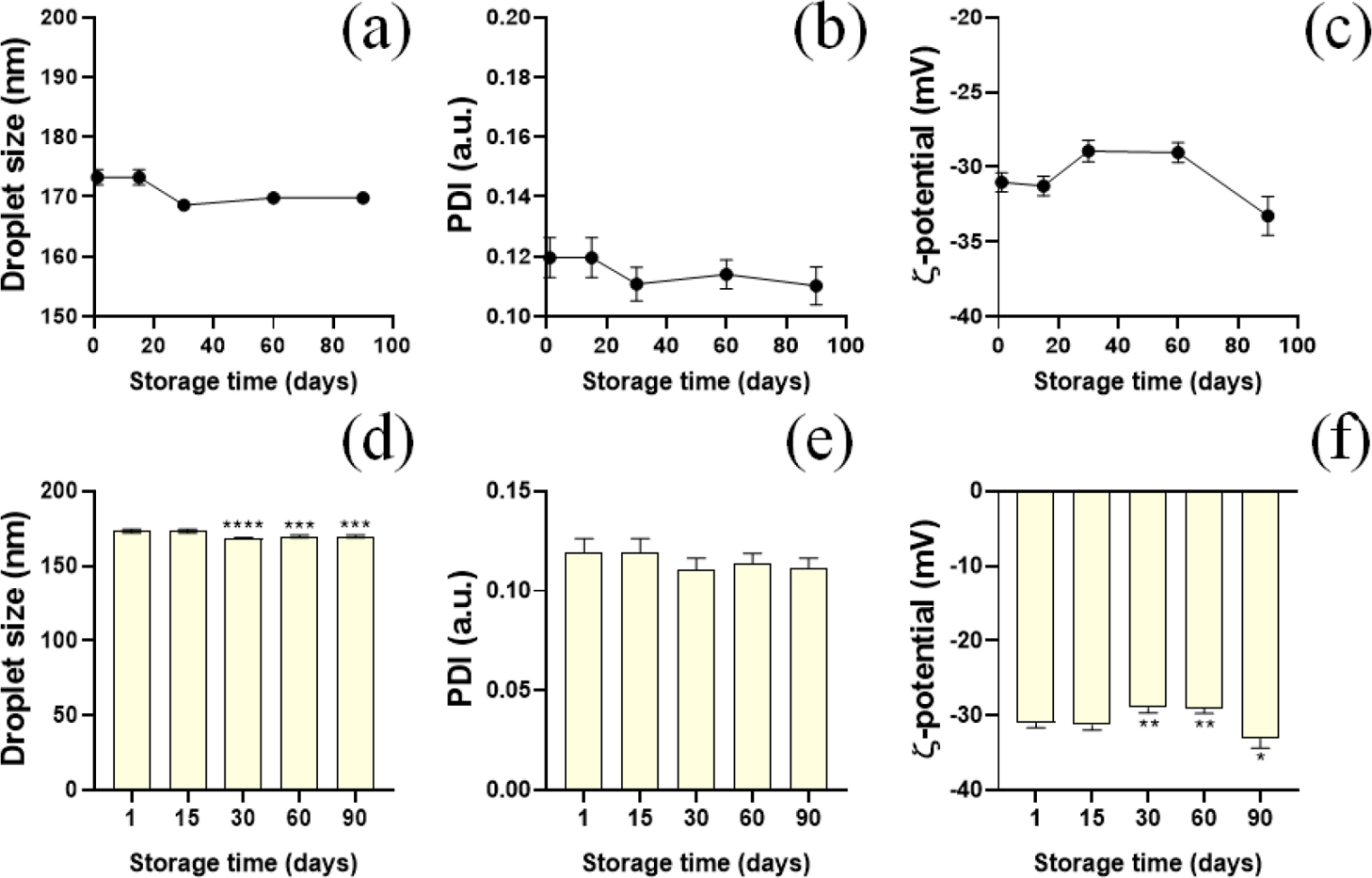
Colloidal properties of carvacrol-loaded nanoemulsion for 90 days at 25 °C. Results obtained of hydrodynamic diameter (nm), PDI and ζ potential (mV) in the storage study of the CNE formulation (a–c); statistical data of the CNE formulation according to the parameters of particle size, PDI and ζ potential in the storage study (d–f).

The ζ-potential is a fundamental electrokinetic parameter for assessing the stability of colloidal suspensions, as it reflects the repulsion arising from interactions between the surface charges of particles.^53^ ζ-potential values close to zero indicate a reduced surface charge, which can promote attractive interactions between particles, making the system more susceptible to destabilization processes such as flocculation and aggregation.^54^ Conversely, colloidal systems considered stable exhibit ζ-potential values greater than |±30| mV, indicating that electrostatic repulsion forces surpass the attractive van der Waals forces.^55^ This condition enhances the system’s resistance to destabilization phenomena, thereby ensuring the long-term stability of colloidal dispersion.

In the present study, the ζ-potential values for the BNE and CNE samples were −32 and −34 mV, respectively, demonstrating stable colloidal systems. The negative signal observed in the analyses is attributed to the adsorption of hydroxyl groups at the oil–water interface, a process linked to hydrogen bonding from the poly­(propylene oxide) and poly­(ethylene oxide) groups present in the structural chains of the Pluronic F127.^56^

The stability of the nanosystems obtained in this study is largely attributed to the use of Pluronic F127 as a surfactant. This polymer promotes electrostatic stabilization through surface charges generated by intermolecular interactions, as well as steric stabilization due to its high molecular weight.^57^ For pharmaceutical applications, steric stabilization is particularly advantageous as it ensures the stability of the colloidal system regardless of the nature of the surface charges, resulting in a more homogeneous and stable dispersion.^17^

### 3.4 Thermodynamic and Storage Stability Studies

For colloidal systems, thermodynamic stability occurs when the system is in equilibrium with its surroundings or at its minimum energy state.^58^ Nanoemulsions, in turn, are colloidal dispersions characterized by predominantly kinetic stability against aggregation processes. In this context, thermodynamic stability assays are designed to identify metastable or intrinsically unstable formulations.^17^ Accordingly, to evaluate the stability of the formulation developed in this study, the carvacrol-loaded nanoemulsion (CNE) was subjected to thermal and mechanical stress tests, including heating–cooling cycles, freeze–thaw cycles, and centrifugation assays.^27^ As expected, the CNE formulation remained visually unchanged throughout the experiments. No visual evidence of phase separation, creaming, precipitation, cracking, or coalescence was observed during the stress stability tests.

In addition to visual inspection, the CNE formulation was subjected to a storage stability study at 25 ± 2 °C for a period of 90 days, with evaluations conducted at intervals of 1, 15, 30, 60, and 90 days. During this period, colloidal stability parameters were analyzed, including particle size, polydispersity index (PDI), and ζ-potential. Figure 2 presents the data obtained from the stability analyses, as well as the statistical treatment applied.

Regarding particle size, it was observed that between 1 and 15 days, there were no statistically significant variations (*p* > 0.05). In the interval between 15 and 30 days, a slight reduction in particle size was observed, ranging from 173 to 169 nm, with statistically significant differences (*p* < 0.0001). Between 30 and 60 days, a slight increase was noted, from 168 to 169 nm (*p* < 0.001), while between 60 and 90 days, the values remained stable, ranging from 170 to 171 nm (*p* < 0.001). Regarding the polydispersity index (PDI), the values remained within the range of 0.11 throughout the analyzed period, with no statistically significant differences between the intervals (*p* > 0.05). As for the ζ-potential, no statistically significant variations were observed between 0 and 15 days (*p* > 0.05). Between 15 and 30 days, a variation was recorded from −32 mV to −29 mV (*p* < 0.01). Between 30 and 60 days, a slight reduction in ζ-potential values was observed, ranging from −29 mV to −30 mV (*p* < 0.01). Finally, between 60 and 90 days, a variation from −29 mV to −33 mV was noted (*p* < 0.05).

The analysis of the data obtained from the thermodynamic and storage stability studies demonstrated that the formulation developed in this study maintained its stability even under conditions that could promote destabilization processes, such as thermal and mechanical stress tests. Furthermore, the values of the physicochemical parameters obtained throughout the storage stability study were similar to those recorded for the freshly prepared sample, indicating that both the CNE formulation process and the excipients used in nanoemulsion preparation were effective in achieving a stable system.^59^

### 3.5 Morphology Analyses

In nanostructured systems, morphology is an essential parameter for assessing stability, as it reflects the relative and cumulative distribution of the size and shape of the suspended droplets. In this context, the present study investigated the morphological aspects of the CNE and BNE formulations, shown in Figure 3, using transmission electron microscopy (TEM) and confocal laser scanning microscopy (CLSM).

**Figure 3:**
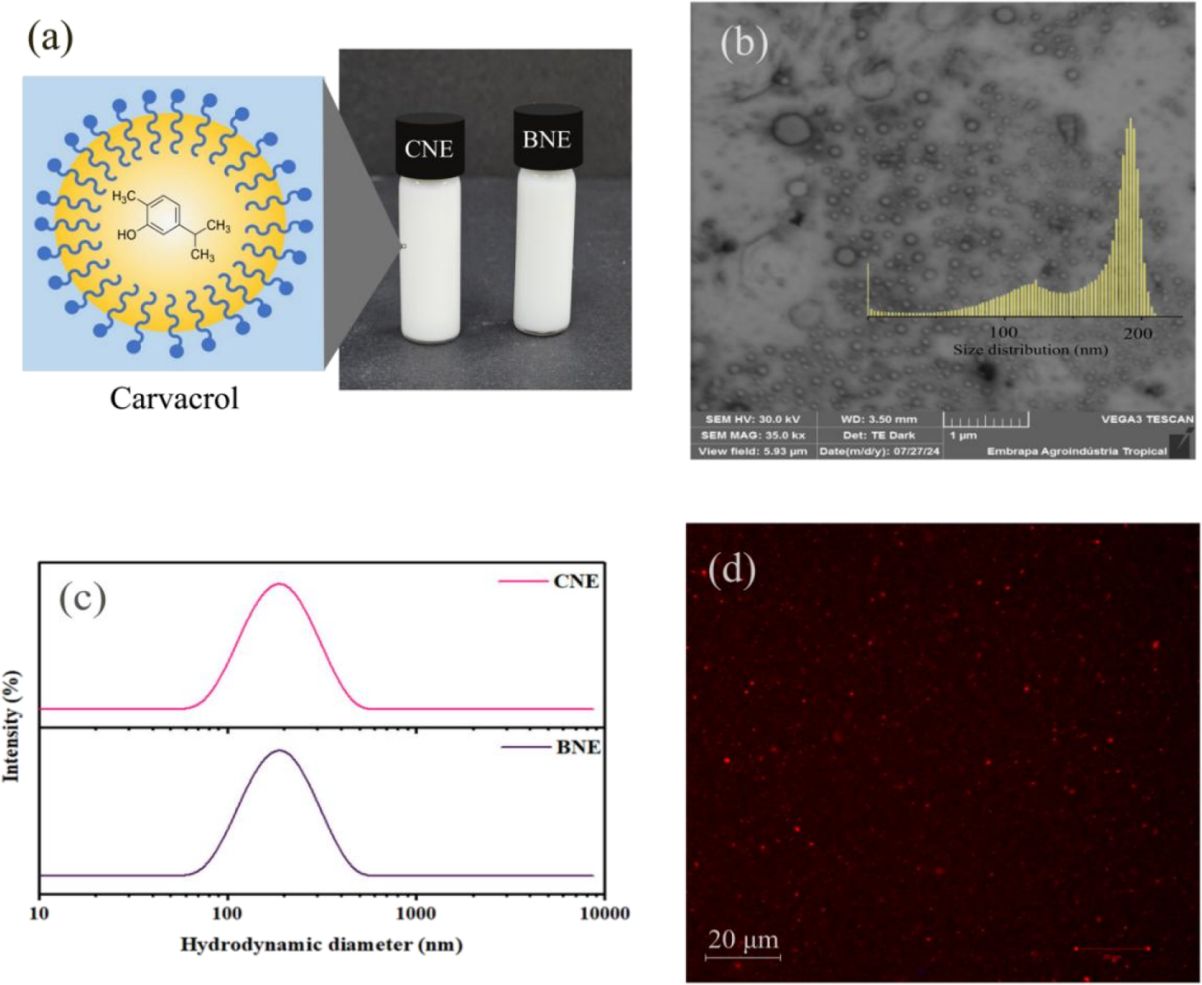
Morphology analyses of synthesized nanoformulations. (a) fresh prepared nanoemulsions (CNE and BNE); (b) morphological characterization by transmission electron microscopy; (c) droplet size distribution of CNE and BNE nanoformulations and (d) morphology assessment by confocal microscopy.

CLSM analyses were performed to investigate possible structural changes in the oil droplets present in the dispersed phase of freshly prepared nanoemulsions stored at ±25 °C. For this purpose, Nile red dye was used, whose fluorescence, emitted when interacting with the organic (oil) phase, allows the visualization of reddish droplets. As shown in Figure 3d, the CNE formulation exhibited small, uniformly distributed droplets, indicating a well-organized dispersed phase. These findings are consistent with previous reports using CLSM to evaluate droplet morphology in essential oil-loaded nanoemulsions.^60^

Additionally, TEM analyses were conducted to evaluate the stability of the obtained nanosystems, with emphasis on the morphology, size, and distribution of suspended particles.^61^ Figure 3b,c presents micrographs and histograms of the CNE sample, revealing particles smaller than 200 nm, with data aligning closely with the results obtained from DLS analyses. The micrographs highlighted spherical droplets with a broad size distribution, characteristic of a moderately polydisperse system.^28^

The formation of nanosized droplets is strongly associated with the high-energy emulsification method employed. Ultrasonic irradiation promotes acoustic cavitation and intense shear forces, resulting in efficient droplet disruption and size reduction.^63^ The presence of Pluronic F127 further contributes to this process by reducing interfacial tension, thereby facilitating nanodroplet formation and stabilization.^18^

Moreover, the incorporation of *A. squamosa* seeds oil (ASSO) into the dispersed phase enhanced the stability of the nanoemulsions. This effect is attributed to the high content of long-chain fatty acids which are known to inhibit Ostwald ripening. The presence of long-chain triacylglycerols in the oil phase is therefore a key factor contributing to the long-term stability of essential oil-loaded nanoemulsion systems.^64^

### 3.6 Encapsulation Efficiency (% EE) and Drug Loading Capacity (% DLC)

The quantification of CARV in the CNE formulation was based on encapsulation efficiency (% EE) and drug-loading capacity (% DLC) values, as presented in Table 2. The developed nanocarriers demonstrated a remarkable ability to incorporate the active compound, achieving % EE values of 99.34 ± 0.06% and % DLC values of 9.93 ± 0.01%. These results are comparable to those reported by Cardoso et al., (2023)^18^ and Li et al., (2025),^65^ who prepared nanoemulsions loaded with carvacrol and obtained EE values of approximately 84% and 94%, respectively. This comparison highlights the efficiency of the preparation method and formulation employed in this study, evidenced by the superior % EE values achieved compared to previously reported data.

The high efficacy observed can be attributed to van der Waals forces arising from intermolecular interactions between carvacrol and the fixed oil from *A. squamosa* L. seeds within the lipophilic core. These interactions are crucial for minimizing the volatilization processes of the active compound and enhancing its resistance to degradation.^66,67^ The high encapsulation efficiency and drug-loading capacity values achieved in this study suggest that these nanocarriers have significant potential to improve the bioavailability of carvacrol, thereby optimizing its biological activities.

### 3.7 In Vitro Drug Release and Kinetic Studies

The *sink* conditions were adjusted to prevent saturation of the bioactive compound in the receptor medium.^25^ To achieve this, carvacrol was subjected to dissolution tests using different proportions of ethanol and phosphate buffer (PBS) at pH 7.4. The addition of ethanol, an organic solvent, proved essential to facilitate carvacrol dissolution due to its low solubility in aqueous media.^68^ The results for the tested proportions are presented in Table 3.

**Table 3:** Results of Solubility Tests and Determination of Sink Conditions

EtOH/PBS (pH 7.4)^a^	Solubility CARV (μg mL^–1^)^b^	Sink condition (μg mL^–1^)
10/90	insoluble	–
20/80	insoluble	–
25/75	1425	≤475
30/70	2426	≤808

a EtOH/PBS (pH 7.4)—ethanol/phosphate-buffered saline pH 7.4.

b CARV: carvacrol.

The data obtained indicates that, among the tested proportions, only the mixtures containing 25% and 30% ethanol and PBS (v v^–1^) were able to solubilize carvacrol, with saturation concentrations of 1425 μg mL^–1^ and 2426 μg mL^–1^, respectively. The sink conditions were defined as 1/3 of the saturation concentration, resulting in values of 475 μg mL^–1^ and 808 μg mL^–1^ for the 25/75 and 30/70 ethanol/PBS proportions, respectively.

In selecting the ideal receptor medium, the results of the encapsulation efficiency test (Table 2) were also considered, in which the CNE formulation exhibited an % EE of 99.34%, corresponding to a mass of 2980 μg of carvacrol per mL of formulation. If 100% of the active compound is released into the receptor medium (12 mL), the maximum concentration achieved would be 248.3 μg mL^–1^, a value lower than the saturation concentrations observed for both tested proportions. Therefore, aiming to minimize the use of ethanol in the receptor medium, the 25/75 ethanol/PBS (pH 7.4) proportion was selected for the release assays. In vitro drug release study was performed with CARV and CNE and the results obtained are presented in Figure 4.

**Figure 4:**
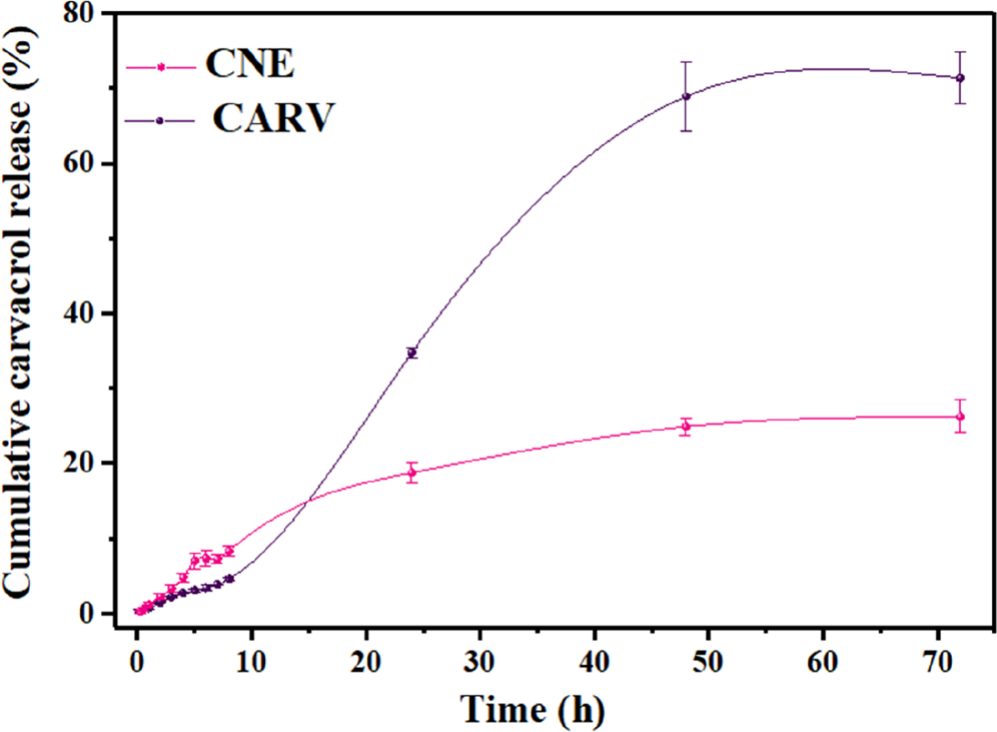
Cumulative release of carvacrol in its free form and encapsulated in the CNE formulation. CNE: carvacrol-loaded nanoemulsion; Carv: free carvacrol.

According to the release assay data, during the first 8 h of the experiment, the diffusion of free carvacrol into the receptor medium occurred at a slightly slower rate compared to that observed for the CNE formulation. This behavior can be attributed to the solubilization of free carvacrol in ethanol, a solvent selected due to the compound’s low aqueous solubility. Despite its hydrophobic nature, carvacrol exhibits a high affinity for polar solvents as a result of dipole–dipole intermolecular interactions, which may have hindered its initial diffusion into the receptor medium.^69^

Nevertheless, these interactions were not sufficient to effectively modulate the diffusion process. By the end of the experiment, 71.4 ± 3.41% (2107 ± 81.6 μg) of free carvacrol had diffused into the receptor medium, whereas the release of carvacrol encapsulated in the CNE formulation plateaued at 26.2 ± 2.2% (781.7 ± 66.3 μg). These results demonstrate that the nanocarriers developed in this study were effective in controlling the diffusion of the bioactive compound, promoting a sustained release of carvacrol throughout the experimental period. These findings are consistent with previous studies highlighting nanoemulsions as promising delivery systems for the encapsulation and controlled release of volatile compounds, owing to their ability to enhance the biological performance of such xenobiotics under physiological conditions.^70^

To investigate the release kinetics of carvacrol-loaded nanoemulsion, the experimental data were fitted to the mathematical models described in the Experimental Section: zero-order, first-order, Higuchi, and Korsmeyer–Peppas. The suitability of the models was evaluated based on the coefficient of determination (*R* ^2^) and the one with the best fit (highest *R* ^2^) described the release mechanism. The values obtained are presented in Table 4.

**Table 4:** Release Kinetic Parameters of Carvacrol Loaded-Nanoemulsion

	Coefficient of determination (*R* ^2^)			
Sample	Zero-order	First-order	Higuchi	Korsmeyer–Peppas
CNE^a^	0.9994	0.9626	0.9832	0.9997

a CNE: carvacrol-loaded nanoemulsion.

The mathematical models that best described the release behavior of the active compound in this study were the zero-order and Korsmeyer–Peppas models, with *R* ^2^ values of 0.9994 and 0.9997, respectively. Zero-order release kinetics state that the drug release process occurs solely as a function of time, maintaining a constant release rate regardless of the initial concentration of the active compound within the nanocarrier.^71^ In zero-order delivery systems, the released drug remains within the therapeutic range throughout the entire lifespan of the nanocarrier.^72^ However, this mathematical model does not consider the structural entrapment of the active compound, making it more applicable to encapsulation systems that do not undergo structural rupture when subjected to contact with the physiological environment.^73^

In the present study, due to the polymeric surface formed by the surfactant Pluronic F127 in the nanosystems, the Korsmeyer–Peppas model was applied to analyze the release of encapsulated carvacrol. This model uses the diffusion exponent (*n*) to classify the release mechanisms in polymer matrices. From the values of *n*, it is possible to determine whether the drug release occurs by diffusion or relaxation mechanisms of the polymer chains. For spherical systems that undergo swelling processes in aqueous medium, the value of (*n*) plays the role of differentiating the release mechanisms of the system under study.^36^

Transport processes whose n values are lower than 0.43 are called case I, indicating that the release follows the Fickian model, and the mechanism used is governed by diffusion processes. In the hypothesis of 0.43 < *n* < 0.85, the transport model is non-Fickian or anomalous, where diffusion and swelling of the polymer chains occur simultaneously.^74^ In the condition of *n* > 0.85, the release is driven by the transport mechanism determined as super case II, which constitutes an extreme form of transport, that is, during the sorption process, tension and relaxation of the polymer chains occur, in addition to this fact, the processes of diffusion and dissolution of the drug in the external environment occur simultaneously.^38^

In the present study, the diffusion coefficient obtained for the CNE formulation was 0.94, which characterizes a super case II mechanism. This result demonstrates the complexity in the carvacrol release process in nanoemulsions, in which nanocarriers act by providing improved control and efficiency in the drug delivery process.

### 3.8 Cytotoxicity and Anti-Inflammatory Potential

The pharmacological potential of nanostructured systems based on ASSO and CARV was carried out based on the analysis of the same parameters (LDH activity and MPO release) used for ASSO, in order to elucidate the effects of the combination between these two natural products, as well as to study the impact of the nanoencapsulation process on preclinical safety and efficacy.

Figure 5 shows the results of the evaluation of the integrity of the cytoplasmic membrane of human neutrophils treated with CARV, CNE and BNE. It was observed that the addition of the vehicle used to solubilize CARV (0.1% DMSO) did not cause significant changes in neutrophil viability (23.3 ± 4.8 U L^–1^) compared to the HBSS group (untreated cells) (24.7 ± 5.8 U L^–1^). In the same way, the treatment with CARV (5–100 μg mL^–1^) did not result in a significant increase in LDH activity (23.8–28.6 U L^–1^) compared to the HBSS group.

**Figure 5:**
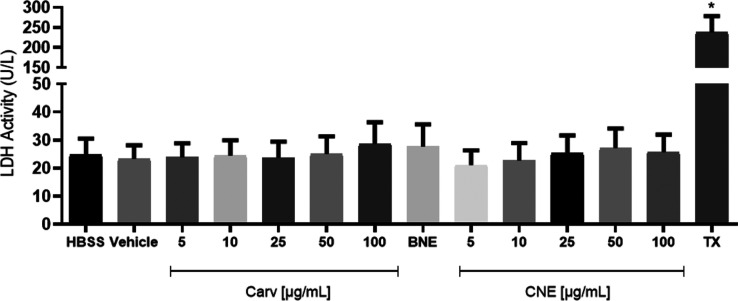


The exposure of these cells to nanostructured systems (CNE and BNE) within the tested concentration range (5–100 μg mL^–1^) did not promote a significant increase in LDH activity (21.1–25.8 U L^–1^) when compared to the control group (HBSS). On the other hand, TX (cytotoxic standard) significantly increased LDH activity (238.0 ± 40.2 U L^–1^) in the extracellular medium, ensuring the effectiveness of the proposed model. These results show that free and nanoencapsulated CARV in ASSO-based emulsions have low cytotoxic potential against human neutrophils. Similar results were reported by Türkez and Aydın (2016),^75^ who evaluated the cytotoxic effect of a carvacrol in human blood cells.

Based on the results of the cytotoxicity assessment, the study of the anti-inflammatory potential continued. Figure 6 illustrates the effect of CARV, CNE and BNE on PMA-induced neutrophil degranulation. The addition of PMA to the neutrophil suspension resulted in a significant increase in MPO release, approximately three times higher compared to untreated cells (HBSS group: 30.08 ± 5.39%). Free carvacrol showed a significant reduction (*p* < 0.05) in MPO release at concentrations of 50 and 100 μg mL^–1^ (56.11 ± 9.29% and 22.82 ± 5.23%, respectively) when compared to the PMA-treated group.

**Figure 6:**
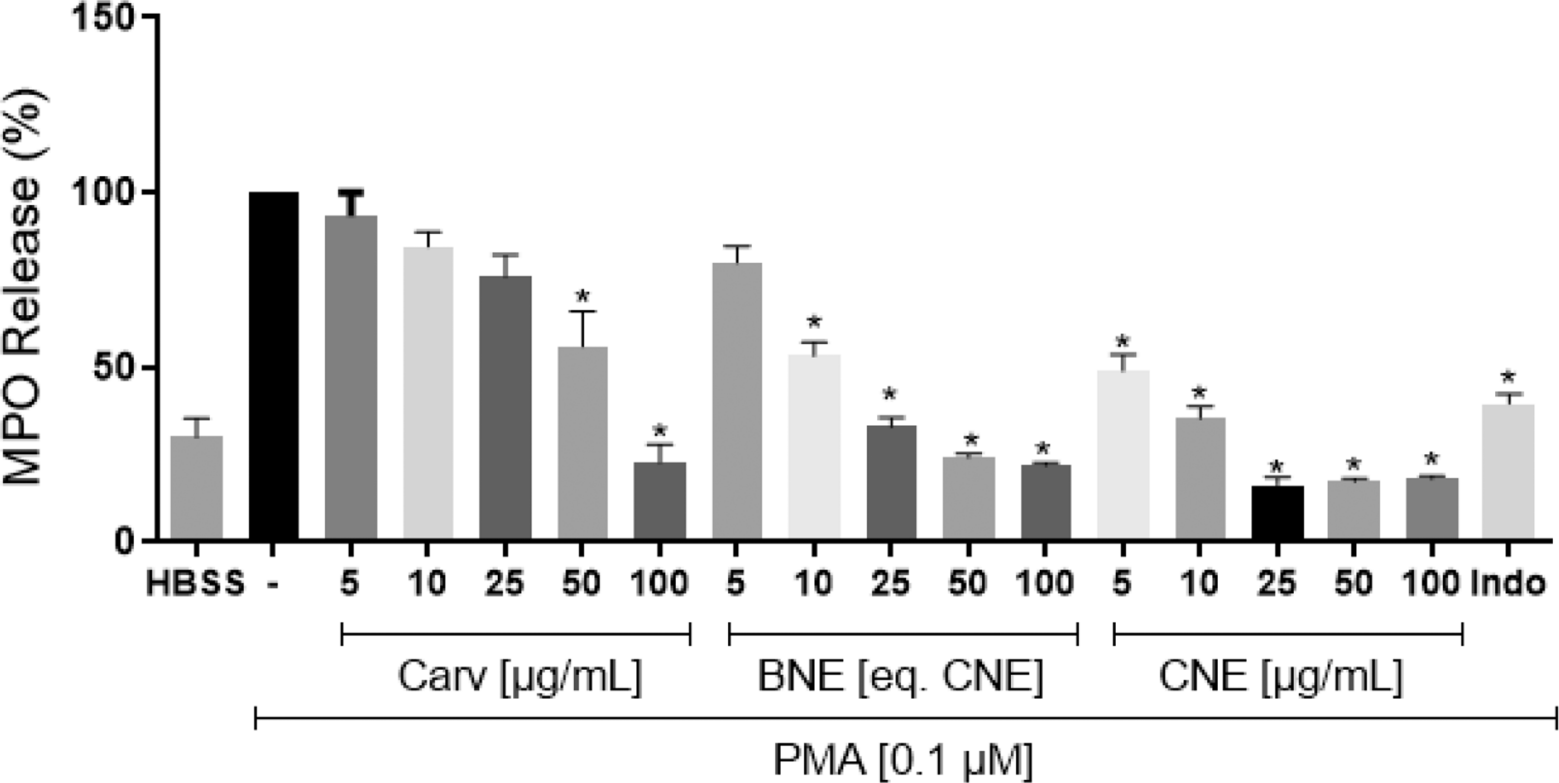
Effect of free carvacrol (CARV), blank nanoemulsion (BNE) and carvacrol-loaded nanoemulsion (CNE) on human neutrophil degranulation assessed by myeloperoxidase enzyme release. Values were expressed as mean ± SEM. Analyses were performed in triplicate. Indo: indomethacin, standard anti-inflammatory drug. Where, *p < 0.05 vs control group (one-way ANOVA followed by Tukey’s post-test).

Additionally, the blank nanoemulsion (BNE) also demonstrated the ability to reduce (significantly) MPO release, although its effect was less pronounced compared to the CNE formulation. At concentrations of 10, 25, 50, and 100 μg mL^–1^, the MPO release values were 53.31 ± 3.92%, 33.05 ± 2.61%, 24.37 ± 0.94%, and 22.12 ± 0.63%, respectively.

The carvacrol-loaded nanoemulsion (CNE), when incorporated into the neutrophil suspension, showed a significant reduction in MPO release, even at lower concentrations, compared to unencapsulated carvacrol. At 5 μg mL^–1^, MPO release was 49.03 ± 4.72%, and at subsequent concentrations of 10, 25, 50, and 100 μg mL^–1^, the observed values were 35.45 ± 3.50%, 16.38 ± 2.30%, 17.45 ± 2.78%, and 18.22 ± 0.76%, respectively.

The effect of CNE on neutrophil degranulation showed a plateau between concentrations of 50 and 100 μg mL^–1^, indicating that the maximum response was reached. This effect is associated with maximum receptor occupancy and saturation of the signaling pathway involved in the pharmacological mechanism.^76^ This behavior has been observed in literature for other natural products, such as the treatment of human neutrophils with the hydroalcoholic extract of the *Agaricus blazei* Murill mushroom.^38^ The hypothesis is that the different bioactive compounds present in the formulation or plant derivative may act on different signaling pathways, contributing to the saturation of the pharmacological effect.

According to the literature, CARV exhibits remarkable anti-inflammatory potential, primarily attributed to its ability to reduce levels of inflammatory mediators such as IL-1β, IL-6, TNF-α and iNOS.^11^ This reduction results in the inhibition of cyclooxygenase-2 production, leading to a decrease in prostaglandin E2 synthesis.^77^ Additionally, CARV possesses antioxidant properties that aid in neutralizing free radicals, thereby promoting tissue protection.^78^

The pharmacological efficacy of CARV was significantly enhanced by the encapsulation process, as evidenced by the superior performance of the CNE formulation compared to the unencapsulated drug. It was also observed that the BNE showed positive results in reducing MPO release, a phenomenon attributed to the inclusion of *A. squamosa* seed oil (ASSO) as a component of the nanoemulsion.

The estimated IC_50_ values for inhibition of neutrophil degranulation by ASSO, CARV, BNE, and CNE were 63.68, 51.71, 14.76, and 3.96 μg mL^–1^, respectively, this result indicates the potential additive effect between the different components of the formulation on anti-inflammatory activity. The effect observed in the CNE formulation can be attributed to the synergism between CARV and ASSO, enabled by the interactions within the lipophilic core of the nanoemulsion. Moreover, the ultrasonic irradiation process employed in the formulation facilitated the formation of nanodroplets, which protect CARV from volatilization and ensure its long-term stability, thereby enhancing its pharmacological effect.^18^ This nanocarrier is also capable of improving the dispersion of ASSO in aqueous media, enhancing its biological effect.

The CNE formulation demonstrated a promising anti-inflammatory effect, with significant reductions in PMA-induced MPO release at all tested concentrations. Although neutrophils play an essential role in immune defense, their excessive or inappropriate activation contributes to the progression of diseases, such as autoimmune conditions and cancer.^79,80^ In this context, the development of safe and effective products for managing inflammatory processes is of great relevance. The ability of CNE to modulate the inflammatory response by reducing MPO release suggests that this formulation could minimize tissue damage and slow the progression of chronic inflammatory diseases, given the critical role of neutrophils in both acute and chronic inflammatory responses.

In addition, this study presented some limitations that prevented a comprehensive analysis of the anti-inflammatory potential of *A. squamosa* seed oil (ASSO) and the prepared nanoemulsions. Among the limitations presented, the following stand out: the use of a single cell type, the absence of in vivo studies, and the lack of molecular tests to understand the mechanism of action involved in the anti-inflammatory effect and no comparison to clinical anti-inflammatory agents beyond indomethacin.

## 4 Conclusion

In the present study, nanoemulsions based on *A. squamosa* L. seed oil were successfully prepared by ultrasonic irradiation to encapsulate and load carvacrol. The nanoformulations exhibited colloidal stability, with particle sizes smaller than 200 nm and spherical morphology, ideal characteristics for nanometric systems. The nanocarriers demonstrated high loading capacity, with encapsulation efficiency exceeding 95%. In vitro drug release assays confirmed the effectiveness of the adopted methodology by prolonging the release profile of carvacrol. The *A. squamosa* L. seed oil, carvacrol and nanocarriers (empty and loaded with carvacrol) presented low cytotoxicity against human neutrophils, since they did not promote the rupture of their cell membranes, which was studied by determining the activity of the enzyme lactate dehydrogenase (LDH). Regarding the anti-inflammatory effect, *A. squamosa* L. seed oil at 50 μg mL^–1^ showed a percentage of MPO release similar to indomethacin (standard anti-inflammatory). Furthermore, encapsulation of carvacrol in the nanostructured system was able to enhance its anti-inflammatory effect, demonstrating a possible synergistic effect between the fixed oil used as organic phase and this bioactive. These results highlight the biological potential of the developed nanoformulations, which enhance the pharmacological effect of carvacrol, offering a promising alternative for the treatment of inflammatory processes.
